# A systematic review of the role of methylase genes in antibiotic resistance: co-existence with extended spectrum β-lactamase and carbapenemase genes in *Klebsiella pneumoniae*

**DOI:** 10.7717/peerj.20428

**Published:** 2025-12-18

**Authors:** Nur Husna Shahimi, Nabiha Bouafia, Mawaddah Mohd Azlan, Asma Nadia Ahmad Faris, Nur Ayunie Ahmad, Ali Rabaan, Amal Alfaraj, Bandar Albaradi, Tasneem Zaidan, Abdulsalam Alawfi, Amer Alshengeti, Zainab Almansour, Wadha Alfouzan, Amal Sabour, Maha Alshiekheid, Anizah Rahumatullah, Nik Yusnoraini Yusof

**Affiliations:** 1 Institute for Research in Molecular Medicine (INFORMM), Health Campus, Universiti Sains Malaysia, Kubang Kerian, Kelantan, Malaysia; 2 Infection Prevention and Control Centre of Excellence, Prince Sultan Medical Military City, Riyadh, Saudi Arabia; 3 Preventive and Community Medicine Department, Faculty of Medicine, University of Sousse, Sousse, Tunisia; 4 Molecular Diagnostic Laboratory, Johns Hopkins Aramco Healthcare, Dhahran, Saudi Arabia; 5 College of Medicine, Alfaisal University, Riyadh, Saudi Arabia; 6 Department of Public Health and Nutrition, The University of Haripur, Haripur, Pakistan; 7 Pediatric Department, Abqaiq General Hospital, First Eastern Health Cluster, Abqaiq, Saudi Arabia; 8 Department of Pediatric Infectious Diseases, King Fahad Specialist Hospital, Dammam, Saudi Arabia; 9 Pediatric Infectious Diseases Unit, Pediatric Department, King Abdulaziz Hospital, Jeddah, Saudi Arabia; 10 Department of Pediatrics, College of Medicine, Taibah University, Al-Madinah, Saudi Arabia; 11 Department of Infection Prevention and Control, Prince Mohammad Bin Abdulaziz Hospital, National Guard Health Affairs, Al-Madinah, Saudi Arabia; 12 Biological Science Department, College of Science, King Faisal University, Hofuf, Saudi Arabia; 13 Department of Microbiology, Faculty of Medicine, Kuwait University, Safat, Kuwait; 14 Microbiology Unit, Department of Laboratories, Farwania Hospital, Farwania, Kuwait; 15 Department of Botany and Microbiology, College of Science, King Saud University, Riyadh, Saudi Arabia

**Keywords:** Extended-Spectrum-Beta-Lactamases, Carbapenemase, *Klebsiella pneumoniae*, 16S rRNA methyltransferase, Extended-Spectrum-β-Lactamases

## Abstract

**Background:**

Antibiotic resistance in multidrug-resistant *Klebsiella pneumoniae* (MDR-KP), particularly against carbapenems and colistin, is a critical concern, increasing morbidity and mortality among hospitalized patients. This systematic review aims to identify methylase genes in *K. pneumoniae* and examine their co-existence with β-lactamase and carbapenemase genes contributing to antibiotic resistance.

**Methods:**

A literature search was conducted across three electronic databases from inception until 6 December 2023. The quality assessment followed Critical Appraisal Skills Programme (CASP) criteria. Studies focusing on methylase genes and antibiotic resistance in *K. pneumoniae* were included. Two authors independently screened titles, abstracts, and full texts, with a third resolving disagreements.

**Results:**

Thirty-four studies met the inclusion criteria. Methylase genes in *K. pneumoniae* isolates were predominantly reported in Europe and Asia, particularly in Iran, China, Japan, and India (8.8%, *N* = 3). The most prevalent 16S rRNA methyltransferase genes identified were *armA* (76.5%, *N* = 26), *rmtB* (61.8%, *N* = 21), and *rmtC* (29.4%, *N* = 10). Common extended-spectrum β-lactamase (ESBL) genes included *bla_CTXM_* (64.7%, *N* = 22) and *bla_SHV_* (47%, *N* = 16), while *bla_KPC_* (26.5%, *N* = 9) and *bla_NDM_* (23.5%, *N* = 8) were the predominant carbapenemase genes. The coexistence of methylase genes with ESBL and carbapenemase genes conferred significant resistance to aminoglycosides (gentamicin, kanamycin, tobramycin, amikacin, arbekacin), cephalosporins (cefazolin, cefoxitin, cefotaxime), and carbapenems (imipenem, meropenem).

**Conclusions:**

The widespread distribution of resistance mechanisms in *K. pneumoniae* highlights a global challenge, emphasizing the need for strategic antimicrobial use to reduce resistance rates.

## Introduction

*Klebsiella pneumoniae* is a prevalent Gram-negative bacterium, accounting for 0.5–5.0% of all pneumonia cases and responsible for approximately 11.8% of all hospital-acquired pneumoniae (Ashurst & Dawson, 2022). It is a leading cause of nosocomial infections, including bacteraemia, septicaemia, meningitis, endocarditis, cellulitis, and urinary tract infections (UTIs). Resultant infections are known to contribute to long-term hospital stay and high mortality rates up to 50%–100% (Ashurst & Dawson, 2022; Esposito et al., 2018; Walter et al., 2018). In Malaysia, a study at Hospital Canselor Tuanku Muhriz Universiti Kebangsaan Malaysia reported 260 cases of *K. pneumoniae* bacteremia with a low mortality rate of 12.3% as compared to other countries such as United States and Italy (Ang et al., 2022; Di Pilato et al., 2021; Magill et al., 2014). This was supported by other study where the carrier rates for *K. pneumoniae* as high as 77% can be observed in the stool of hospitalized patients, correlating with antibiotic exposure (Ashurst & Dawson, 2022).

The rise of multidrug-resistant *K. pneumoniae* (MDR-KP) poses a major challenges as current potent antibiotics such as carbapenems and colistin are becoming less effective against resistant strains (Omeershffudin & Kumar, 2022; Giordano et al., 2018; Li et al., 2022). Resistant to the antibiotics have severe implications for patient’s morbidity, mortality and health care burden (Franco et al., 2009). The WHO and other organizations have formulating action plans to mitigate antimicrobial resistance (AMR) through infection prevention strategies, optimize antimicrobial therapies, innovate novel pharmaceuticals and diagnostic methodologies. Despite these efforts have significantly contributed to AMR prevention, the issue remains persistent, continually threatening the efficacy of antibiotics in infection treatment (Yamin et al., 2023), necessitating further research into resistance mechanisms and novel therapeutic strategies.

Despite from extensive research on antibiotics from recently published studies, the role of methylation towards multiple antibiotic resistance has not adequately clarified. Methylation is the process of epigenetics where it involves modifications of DNA bases, histone proteins, and/or non-coding-RNA biogenesis, affecting gene expression without altering nucleotide sequence (Kumar et al., 2017). Several previously published studies suggest that selective pressures induced by non-lethal antibiotic concentrations affect methylation and antibiotic resistance phenotypes, which may affect epigenetic modification of the bacterial genome and facilitate the development of antibiotic resistance *via* gene transfer (Yuan et al., 2021; Ghosh et al., 2020). Further research is needed to elucidate this relationship and identify alternative ways to inhibit multidrug resistance, indirectly assisting healthcare systems in treating patients with *K. pneumoniae* infections. Therefore, this systematic review aims to identify methylase genes in *K. pneumoniae* and examine their co-existence with β-lactamase and carbapenemase genes contributing to antibiotic resistance.

## Materials & Methods

This systematic review followed Preferred Reporting Items for Systematic Reviews and Meta-analyses 2020 (PRISMA) statement (Page et al., 2021) (refer to Supplementary File). The review process involved three-step methods as previously described in Shahimi et al. (2022), particularly identification of the keywords through the databases, screening of the articles based on tittle or abstracts and full text articles assessed for eligibility.

### Search strategy and terminology

A comprehensive search was conducted in National Library of Medicine (PubMed), PubMed Central^®^ (PMC) and Web of Science (WoS) from inception until 6 December 2023. The search included studies published in English using the terms: “methylation”, OR “methylase genes” AND “antibiotic resistance”, OR “multidrug resistant” OR “antibiotic susceptibility” OR “drug resistant” AND “*Klebsiella pneumoniae*”. Duplicates were eliminated across databases. Two authors (NHS and NYY) independently screened titles, abstracts and full-text articles of the potentially eligible studies. Any disagreements were resolved by consensus or discussion with a third reviewer (NAB), who served as the referee. Additional articles were identified by checking the reference lists of full-text articles included in the systematic review.

### Inclusion and exclusion criteria

Inclusion criteria were: (1) studies that investigated methylation in *K. pneumoniae*; (2) studies that accessed the effect of methylation towards antimicrobial resistance. Exclusion criteria included studies that did not focus on methylation, antimicrobial resistance, and *K. pneumoniae*. Furthermore, review articles, editorials, reports, commentaries, conference abstract, unpublished studies and book chapters were excluded to ensure that all relevant studies were included. There were no restrictions on study location, specimen collection date and year of publication.

### Quality assessment

Risk of bias was evaluated using the Critical Appraisal Skills Programme (CASP) checklist (Critical Appraisal Skills Programme (CASP), 2018). Seven key criteria were adapted to this review’s context: clarity of research aims, appropriateness of study design, description of detection methods, replication feasibility of index tests, clear definition of positive results, robustness of analysis, and reporting of ethical approval. Each study was scored for each criterion as met or not met. Scores were summarized to reflect overall quality. Discrepancies were addressed through consensus discussions between the two reviewers (NHS, NYY), with complete agreement achieved on the risk of bias assessment, as described by Shahimi et al. (2022).

### Data extraction and synthesis

Identified included studies were imported into a reference management software (EndNote Version X9; Clarivate Analytics) for duplicate removal. Data were extracted by one author (NHS) and crossed checked by a second author (NYY). A standardized data extraction form was developed to collect key information from each included study. Data items included first author, publication year, country of study, sample size, clinical specimen source, detection methods for methylase genes, types of methylase, ESBL and carbapenemase genes identified, antibiotic susceptibility profiles, and reported minimum inhibitory concentrations (MICs) where available. Extraction was performed by one author and independently verified by a second reviewer to ensure accuracy. Due to the heterogeneity of study designs, detection methods, and outcomes, a narrative synthesis approach was used. Findings were grouped by geographic region, detection technique, gene prevalence, co-occurrence patterns, and antimicrobial resistance profiles. Where possible, frequencies and percentages were calculated to illustrate common patterns across studies.

## Results

### Description of studies

**Figure 1:**
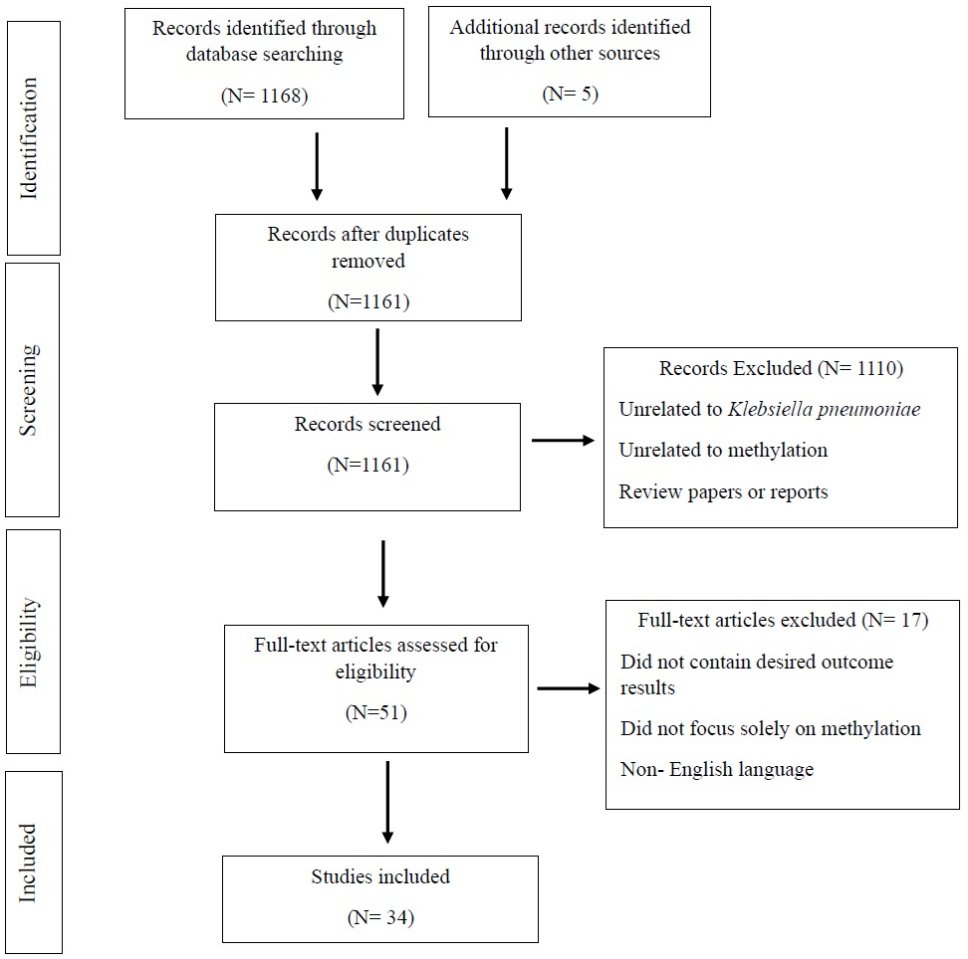
A PRISMA flow diagram illustrates the study selection process and literature search results. Three major databases, PubMed (National Library of Medicine), PubMed Central^®^ (PMC), and Web of Science (WoS) were systematically searched using predefined strategies to identify studies reporting on methylase genes in antibiotic resistance. A total of 1,161 records were retrieved, and duplicates. The remaining records were then screened based on predefined inclusion criteria before being selected for this systematic review.

A total of 1,173 records were retrieved through the database search. After removing 12 duplicates, 1,161 titles and abstracts were screened. Following the application of inclusion and exclusion criteria, 34 studies were included in the final review. The full selection process is illustrated in the PRISMA flow diagram (Fig. 1).

### Risk of bias

**Figure 2:**
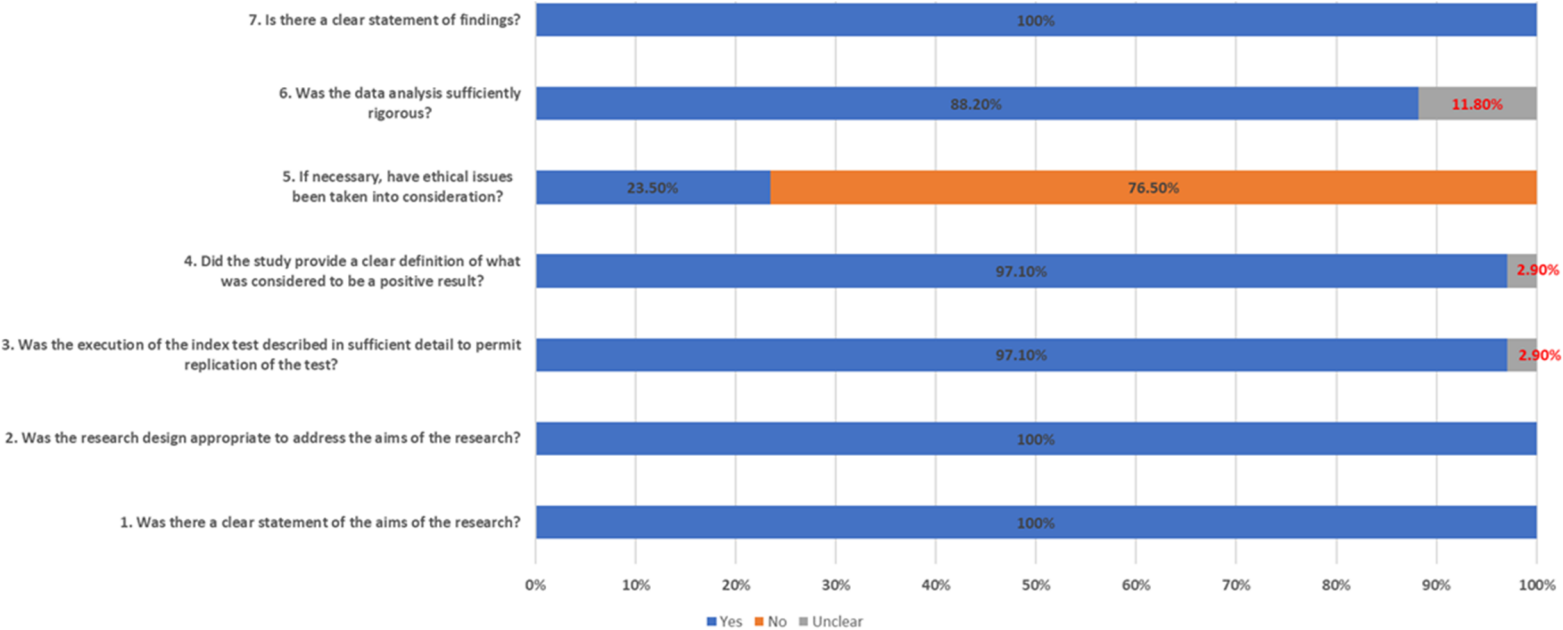
Quality assessment of included studies.

Table S1 and Fig. 2 summarises the quality assessment of included studies (*n* = 34). Most studies clearly outlined their research aims, appropriate research design, execution of the index test which enable replication of the test, clear definition of the positive results and rigorous data analysis. However, only eight studies (23.5%) took into consideration ethical issues in their works.

### Characteristics of included studies

#### Sample size and sources of clinical isolates

The total number of *K. pneumoniae* isolated in the included articles was summarized in Table S2. Among the 34 included studies, *K. pneumoniae* isolates ranged from one (O’Hara et al., 2013) to 502 samples (Taylor et al., 2018). Urine was the most common source (41.2% in 14 out of 34 studies), followed by blood (26.5% in nine out of 34 studies), surgical wounds (20.6% in seven out of 34 studies), sputum (17.6% in six out of 34 studies) and respiratory samples (14.7% in five out of 34 studies). Additional sources included such as pus (11.8%), skin or soft tissue (8.8%), faecal (5.9%), trachea (5.9%), gastrointestinal (3%), abscess (3%), body fluids (3%), ulcer (3%), peritoneal and cerebrospinal fluid (3%), bile fluid (3%) and endotracheal aspirate secretions (3%).

#### Geographical distribution of methylase genes

**Figure 3:**
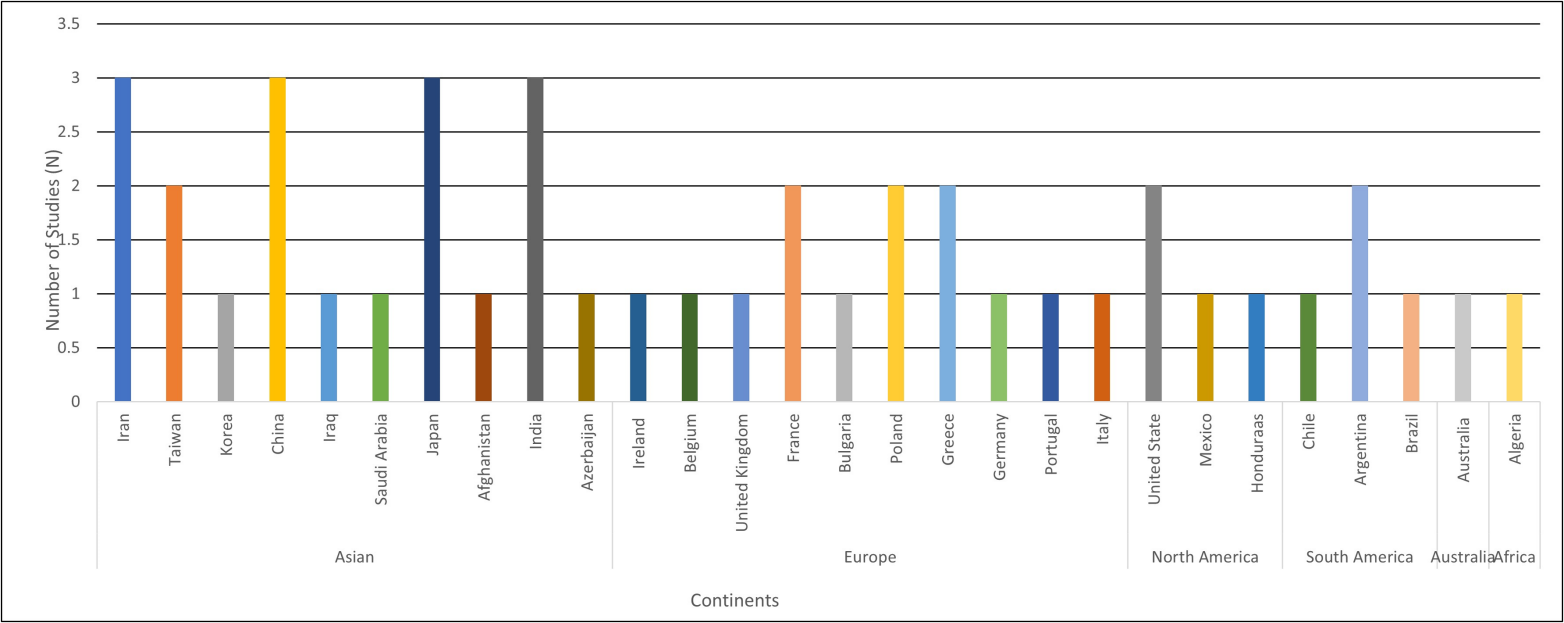
Distribution of methylase genes across countries. Geographic distribution of studies reporting methylase genes in *Klebsiella pneumoniae* across different countries and continents. Most studies were conducted in Asia, particularly Iran, Japan, and China, while Europe, North America, South America, Australia, and Africa contributed fewer studies.

Figure 3 reported methylase genes were detected in 28 countries, predominantly in European (*N* = 10 countries, 35.7%, and Asian (*N* = 10 countries, 35.7%) countries (Costello et al., 2019). European reports included Ireland (Taylor et al., 2018), Belgium (Bogaerts et al., 2007), United Kingdom (Taylor et al., 2018), France (Fritsche et al., 2008; Galimand, Courvalin & Lambert, 2012), Bulgaria (Sabtcheva et al., 2008), Poland (Zacharczuk et al., 2011; Piekarska et al., 2016), Greece (Galani et al., 2011; Nafplioti et al., 2021), Germany (McGann et al., 2016), Portugal (Spadar et al., 2021) and Italy (Sacco et al., 2022). Meanwhile, Asian studies originated from Iran (Pakzad et al., 2019; Yeganeh Sefidan, Mohammadzadeh-Asl & Ghotaslou, 2019; Ahmadian Alashti & Ghane, 2020), Iraq (Ghosh et al., 2020), Saudi Arabia (Al Sheikh et al., 2014), Afghanistan (McGann et al., 2016), Azerbaijan (Yeganeh Sefidan, Mohammadzadeh-Asl & Ghotaslou, 2019), Taiwan (Yan et al., 2004; Ma et al., 2009), Korea (Lee et al., 2006), China (Wu et al., 2009; Yu et al., 2009; Shen et al., 2020), Japan (Nagasawa et al., 2014; Oshiro et al., 2015; Ishizaki et al., 2018), and India (Wangkheimayum et al., 2017; Gopalakrishnan et al., 2018; Tipparthi et al., 2020). Moreover, methylase genes were also reported in Australia (*N* = 1 country; 3.57%) (Guo et al., 2014) and Algeria (*N* = 1 country, 3.57%) (Belbel et al., 2014). On the other hand, North America accounted for 10.7% (Costello et al., 2019) with methylase genes specifically distributed among the United State (Fritsche et al., 2008; McGann et al., 2016), Mexico (Fritsche et al., 2008) and Honduras (McGann et al., 2016), while the remaining three studies in South America (*N* = 3 countries, 10.7%) including Chile (Fritsche et al., 2008) and Argentina (Fritsche et al., 2008; Tijet et al., 2011; Roch et al., 2021).

#### Detection methods for 16S rRNA methyltransferase (16S RMTase)

Detection methods for 16S RMTase were summarized in Table S2. Majority of the included studies (79.4%, *N* = 27 studies) utilized polymerase chain reaction (PCR) technique, with real-time PCR employed in two studies (5.9%) (McGann et al., 2016; Guo et al., 2014). In addition, eleven studies (32.4%) employed sequencing methods to detect methyltransferase genes including whole genome sequencing (WGS) (Taylor et al., 2018; Sacco et al., 2022; Roch et al., 2021), PacBio sequencing (Spadar et al., 2021) as well as Sanger sequencing (Shen et al., 2020). Nine studies (26.5%) combined both PCR and sequencing techniques to detect 16S RMTase (Yan et al., 2004; Wu et al., 2009; Yu et al., 2009; O’Hara et al., 2013; Piekarska et al., 2016; Taylor et al., 2018; Costello et al., 2019; Shen et al., 2020; Sacco et al., 2022). In addition to commonly used PCR and sequencing techniques, Nagasawa et al. (2014) and Oshiro et al. (2015) employed loop-mediated isothermal amplification (LAMP) and immunochromatographic assays using novel monoclonal antibodies (mAbs) respectively. Only one study directly introduced methyltransferase genes onto *K. pneumoniae* strains without additional detection test (Ishizaki et al., 2018).

### The prevalence of methylase genes and aminoglycoside resistance

**Figure 4:**
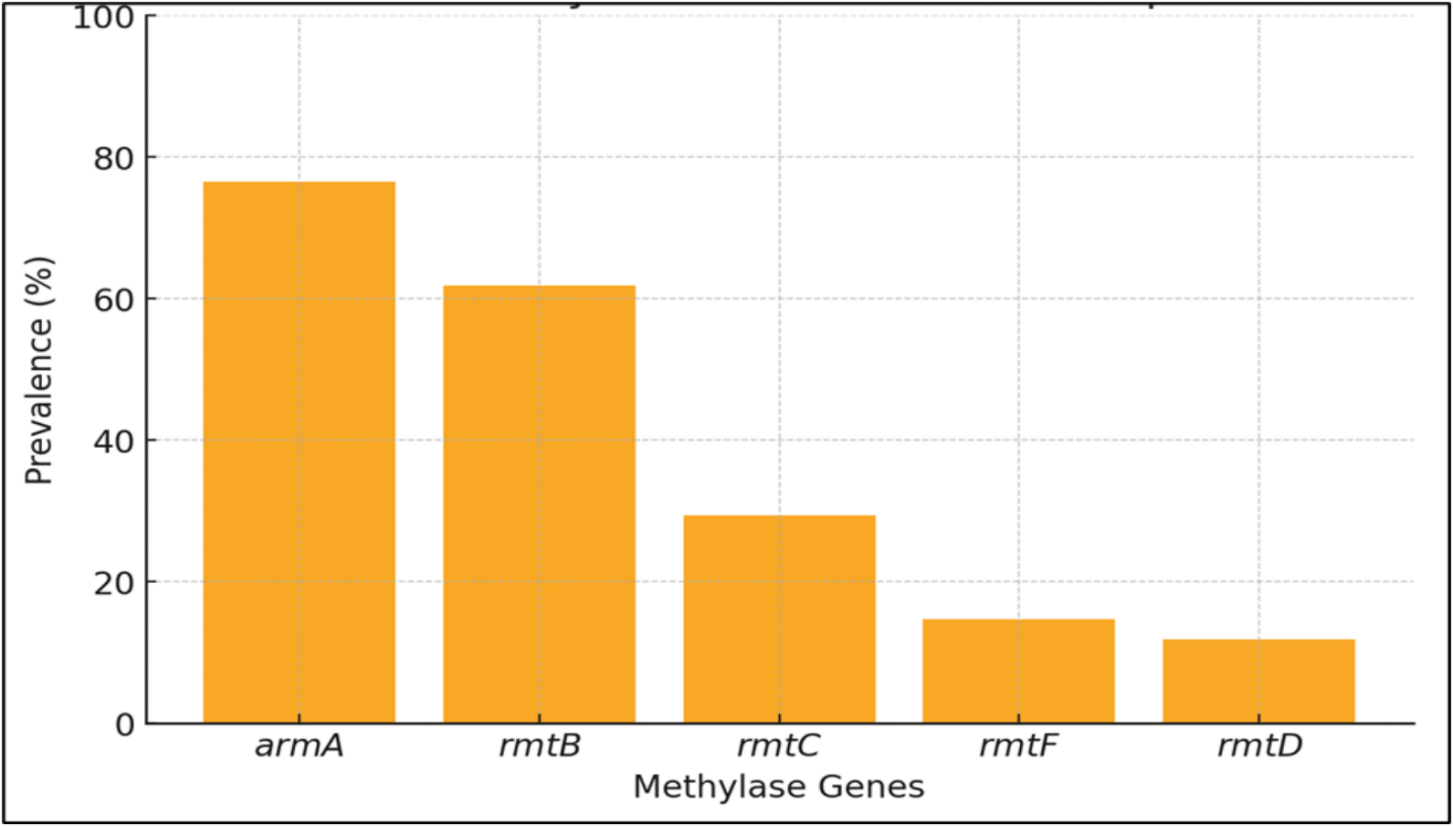
Prevalence of methylase genes in *Klebsiella pneumoniae*. Among the genes identified, *armA* had the highest prevalence, followed by *rmtB*. The genes *rmtC*, *rmtF*, and *rmtD* were detected less frequently.

RMTases can be classified as those producing N7-methyl G1405 such as RmtA, RmtB, RmtC, RmtD1, RmtD2, RmtE, RmtF, RmtG, RmtH and ArmA (Ahmadian Alashti & Ghane, 2020). Among the 16S RMTases genes observed and illustrated in Fig. 4, *armA* gene was the most prevelent (76.5%, *N* = 26), followed by *rmtB* (61.8%, *N* = 21), *rmtC* (29.4%, *N* = 10), *rmtF* (14.7%, *N* = 5) and *rmtD* (11.8%, *N* = 4) genes. Three studies observed the presence of *rmtA* gene (8.82%) in a significant proportion of *K. pneumoniae* (Bogaerts et al., 2007; Galimand, Courvalin & Lambert, 2012; Ahmadian Alashti & Ghane, 2020). Two studies (5.88%) detected *rmtE* (Galimand, Courvalin & Lambert, 2012; Ahmadian Alashti & Ghane, 2020) and *rmtH* genes (O’Hara et al., 2013; McGann et al., 2016) respectively, whereas only one study observed the presence of *Kmr* gene (Galimand, Courvalin & Lambert, 2012). In addition to that, *NpmA* gene, a RMTases that produce N1-methyl A1408 was discovered by a small number of studies (5.88%, *N* = 2) (Al Sheikh et al., 2014; Ishizaki et al., 2018).

Several included studies identified *rmtD, rmtB*, and *rmtF* subtypes, particularly *rmtD1* gene in one study by Tijet et al. (2011) and *rmtB1*, *rmtB4*, and *rmtF1* genes in another study (2.9%) by Costello et al. (2019). Co-existence of multiple RMTAse genes further complicated the treatment, with *armA* and *rmtC* genes detected in two studies (5.88%) (Al Sheikh et al., 2014; McGann et al., 2016), one study (2.9%) observed the combination of *rmtC* and *rmtF* (Oshiro et al., 2015) and *armA* and *rmtB* genes occurred in four of the studies (11.7%) (Ma et al., 2009; Yu et al., 2009; Al Sheikh et al., 2014; Shen et al., 2020). Furthermore, *K. pneumoniae* carrying 16S RMTases in this study exhibited resistance to the 4,6-disubstituted 2-deoxystreptamine (DOS) group of aminoglycosides, mainly gentamicin, kanamycin, tobramycin, amikacin, and arbekacin. However, some studies show that the bacteria are also capable of being resistant to netilmicin (Bogaerts et al., 2007; Fritsche et al., 2008; Galani et al., 2011; Pakzad et al., 2019), neomycin (Piekarska et al., 2016), fortimicin (Fritsche et al., 2008), apramycin (Ishizaki et al., 2018; Nafplioti et al., 2021), streptomycin (Wangkheimayum et al., 2017) and fosfomycin (Spadar et al., 2021).

### Types of resistance genes and antimicrobial resistance

A total of 26 included studies (76.5%) identified carbapenemase and ESBL genes in *K. pneumoniae* isolates co-producing with 16S RMTases. In Table S2, we observed that *bla*_*CTXM*_ (Ma et al., 2009; Wu et al., 2009; Yu et al., 2009; Tijet et al., 2011; Belbel et al., 2014; Wangkheimayum et al., 2017; Gopalakrishnan et al., 2018; Pakzad et al., 2019; Shen et al., 2020; Nafplioti et al., 2021) and *bla*_*SHV*_ (Ma et al., 2009; Yu et al., 2009; Tijet et al., 2011; Zacharczuk et al., 2011; O’Hara et al., 2013; Belbel et al., 2014; Gopalakrishnan et al., 2018; Pakzad et al., 2019; Shen et al., 2020) are the most predominant ESBL genes, meanwhile, *bla*_*KPC*_ (Tijet et al., 2011; McGann et al., 2016; Taylor et al., 2018; Shen et al., 2020; Tipparthi et al., 2020; Nafplioti et al., 2021) and *bla*_*NDM*_ (McGann et al., 2016; Wangkheimayum et al., 2017; Taylor et al., 2018; Shen et al., 2020) were the dominant carbapenemase genes.

### Extended-spectrum β-lactamases (ESBL) genes

Among 22 studies (64.7%) that detected *bla*_*CTXM*_, four studies (18.2%) (Yan et al., 2004; Lee et al., 2006; Bogaerts et al., 2007; Al Sheikh et al., 2014) observed *bla*_*CTXM-14*_ and *bla*_*CTXM-3*_ (Yan et al., 2004; Bogaerts et al., 2007; Sabtcheva et al., 2008; Zacharczuk et al., 2011) variant in *K. pneumoniae* respectively, meanwhile three studies (13.6%) observed *bla*_*CTXM-15*_ (Bogaerts et al., 2007; O’Hara et al., 2013; Spadar et al., 2021) and only one study (4.5%) observed *bla*_*CTXM-1*_ (Sabtcheva et al., 2008). On the other hand, among the 16 studies (47%) identifying bla_SHV_, four studies (25%) observed the presence of *bla*_*SHV-1*_ (Yan et al., 2004; Bogaerts et al., 2007; Sabtcheva et al., 2008; Wu et al., 2009), two studies (12.5%) observed *bla*_*SHV-12*_ (Wu et al., 2009; Al Sheikh et al., 2014) and one study (6.25%) observed *bla*_*SHV-71*_ (Sabtcheva et al., 2008). *bla*_*TEM*_ genes were detected in 14 studies (Tijet et al., 2011; Zacharczuk et al., 2011; Belbel et al., 2014; Guo et al., 2014; Wangkheimayum et al., 2017; Gopalakrishnan et al., 2018; Pakzad et al., 2019; Nafplioti et al., 2021) with *bla*_*TEM-1*_ (Yan et al., 2004; Sabtcheva et al., 2008; Wu et al., 2009; Yu et al., 2009; Al Sheikh et al., 2014) and *bla*_*TEM-3*_ (Bogaerts et al., 2007) found in five and one study respectively. Lastly, variants of *bla*_*OXA*_ which are *bla*_*OXA-10*_ (Galani et al., 2011; Guo et al., 2014; Nafplioti et al., 2021), *bla*_*OXA-1*_ (Galimand, Courvalin & Lambert, 2012; O’Hara et al., 2013; Spadar et al., 2021) *bla*_*OXA-30*_ (Guo et al., 2014), *bla*_*OXA-2*_ (Wangkheimayum et al., 2017) were also found in three, three, one and one studies respectively.

### Carbapenemase genes

Generally, among *bla*_*KPC*_-positive isolates (26.5%), two studies (22.2%) (Galani et al., 2011; Roch et al., 2021) observed *bla*_*KPC-2*_ variant and one study (11.1%) (Spadar et al., 2021) observed *bla*_*KPC-3*_ variant respectively. On the other hand, half of the studies (*N* = 4) that detected *bla*_*NDM*_ showed a variant of *bla*_*NDM-1*_ in the *K. pneumoniae* isolates (Galimand, Courvalin & Lambert, 2012; Guo et al., 2014; Gopalakrishnan et al., 2018; Sacco et al., 2022). Additionally, three studies (27.3%) detected *bla*_*OXA-48*_ (Taylor et al., 2018; Tipparthi et al., 2020; Nafplioti et al., 2021), whilst four and two studies detected *bla*_*VIM*_ (Gopalakrishnan et al., 2018; Taylor et al., 2018; Tipparthi et al., 2020; Nafplioti et al., 2021) and *bla*_*IMP*_ (Gopalakrishnan et al., 2018; Shen et al., 2020).

### Antimicrobial resistance in association with ESBL and carbapenemase genes

The co-production of ESBL and carbapenemase genes had significantly increased resistance to cephalosporin and carbapenems, limiting treatment for *K. pneumoniae* infections. Ma et al. (2009) reported that *K. pneumoniae* isolates were resistant to first generation cephalosporin which is cefazolin with minimum inhibitory concentration (MICs) 32 mg/ml. Meanwhile three studies reported resistance to the second generation cefoxitin (Lee et al., 2006; Belbel et al., 2014; Shen et al., 2020). Resistance to third generation cephalosporin was widespread, including cefotaxime (MICs, 16–256 mg/ml) (Yan et al., 2004; Bogaerts et al., 2007; Sabtcheva et al., 2008; Ma et al., 2009; Belbel et al., 2014; Pakzad et al., 2019; Shen et al., 2020; Tipparthi et al., 2020; Sacco et al., 2022), ceftazidime (MICs, 4–256 mg/ml) (Yan et al., 2004; Bogaerts et al., 2007; Ma et al., 2009; O’Hara et al., 2013; Belbel et al., 2014; Wangkheimayum et al., 2017; Pakzad et al., 2019; Shen et al., 2020; Tipparthi et al., 2020; Sacco et al., 2022), ceftriaxone (O’Hara et al., 2013; Wangkheimayum et al., 2017; Tipparthi et al., 2020) and cefixime (Wangkheimayum et al., 2017). As for fourth generation cephalosporin, six studies reported resistance to cefepime with MICs, 4 to 64 mg/ml (Yan et al., 2004; Bogaerts et al., 2007; O’Hara et al., 2013; Wangkheimayum et al., 2017; Shen et al., 2020; Sacco et al., 2022).

Carbapenem resistance was also significant, with six studies reporting imipenem resistance (Lee et al., 2006; Al Sheikh et al., 2014; Shen et al., 2020; Tipparthi et al., 2020; Spadar et al., 2021; Sacco et al., 2022) and five reporting meropenem resistance (Lee et al., 2006; Al Sheikh et al., 2014; Shen et al., 2020; Tipparthi et al., 2020; Sacco et al., 2022). These resistance mechanisms are primarily mediated by β-lactamase enzymes encoded by genes such as *bla*_*CTX- M*_, *bla*_*SHV*_, and *bla*_*TEM*_ which hydrolyze β-lactam ring. Additionally, carbapenem-resistant *K. pneumoniae* strains harbour additional β-lactamase genes such as *bla*_*KPC*_, *bla*_*NDM*_, and *bla*_*OXA-48*_, further reducing treatment efficacy. A detailed summary of antibiotic resistance was provided in Table S3.

## Discussion

The findings from this study underscore the significant prevalence and implications of 16S rRNA methyltransferase (16S RMTases) in antibiotic resistance, particularly in relation to aminoglycosides, extended-spectrum β-lactamases (ESBL) and carbapenemase. Among the identified 16S RMTases genes, *armA* and *rmtB* were the most prevalent in *K. pneumoniae* isolates, serving as a key mediator of aminoglycoside resistance. The co-occurrence of ESBL and carbapenemase genes further complicates the treatment as these genes confer resistance to a broad range of β-lactam antibiotics, particularly cephalosporin and carbapenems. These findings warrant an urgent need for robust antibiotic surveillance and targeted control measures to mitigate the spread of MDR pathogens.

*K. pneumoniae* is primarily a human pathogen, accounts for 3% to 8% of nosocomial infection or hospital-acquired infections (HIAs) (Ashurst & Dawson, 2022) and therefore has a lower detection rate in environmental sources than in clinical samples. The pathogens mainly colonize mucosal surfaces such as respiratory tract (Chang et al., 2021), patient’s gastrointestinal tract as well as the hospital personnel, which explained its high prevalence in urine, blood samples, surgical wound, sputum and respiratory observed in these studies. Similar case was observed in Malaysia (Ang et al., 2022), where 260 cases of *K. pneumoniae* bacteraemia with 12.3% mortality rate reported. On the other hand, 77% of hospitalized patients carried *K. pneumoniae* in their stool and frequently associated with the antibiotics administered (Ashurst & Dawson, 2022). Proper antibiotic stewardship is crucial in reducing resistance rates and minimized morbidity and mortality.

The highest incidence of *K. pneumoniae* infections was found in Asian and European continents, consistent with previous studies showing colonization rates of 18.8% to 87.7% in Asia and 5% to 35% in Western countries (Lin et al., 2012; Russo & Marr, 2019). Dense urbanization, population density, global travel, trade and migration could be contributor to the spread of infections (Church, 2004; Baker et al., 2022; Relman, Choffnes & Mack, 2013). Antibiotic resistance genes spread among the hosts through the mechanism of horizontal gene transfer (HGT), a phenomenon that is frequently surged in a densely populated city areas with extensive antibiotic usage (Jian et al., 2021; Barathe et al., 2024). This emphasises the importance of coordinated global surveillance and control efforts to prevent further dissemination.

Notably, the findings from this study revealed that *K. pneumoniae* pathogens employed multiple resistance mechanisms including methylase genes production, β-lactamases, and carbapenemases, enabling it to evade antimicrobial action (Jian et al., 2021). Methylase genes play a crucial role in inhibiting protein synthesis by preventing the binding between 30S and 50S ribosomal RNA subunit. The mechanism involved the methylation of specific sites in ribosomal RNA where the added methyl groups will alter the structural conformation of these RNA subunits. Structural studies have shown that RMTases use their N-terminal domain to dock onto the helices of the 30S subunit, while the C-terminal catalytic domain binds to the cofactor S-adenosylmethionine (SAM) and transfer the methyl group to specific nucleotides of G1405 or A1408 within helix 44 for methylation (Srinivas et al., 2023). As a result, the methylation will hinder the interaction or binding between the ribosomal subunit and messenger RNA (mRNA) (Zhang et al., 2023). The methylation on the specific binding site will significantly reduce the ability of the drugs to bind to the specific site of ribosome, indirectly reduce the drug’s efficacy (Moore, Le & Fan, 2013). The methylation of 16S rRNA not only blocks aminoglycoside binding but also limits the effectiveness of combination therapies that rely on synergistic ribosomal inhibition. These findings support the development of new generations of aminoglycosides or adjuvant compounds that can bypass or inhibit methylation pathways, as has been attempted with plazomicin, a next-generation aminoglycoside shown to retain activity against certain 16S RMTase-producing strains (Shaeer et al., 2019). Further research into novel ribosome-targeting antimicrobials or inhibitors that disrupt methylase enzyme function could present alternative therapeutic avenues.

Moreover, presence of enzyme ESBL leads to resistance to first-to-third generation cephalosporins, aztreonam (but not cephamycin or carbapenem). The mechanism of resistance involves the hydrolysis of β-lactam antibiotics, which occurs when the enzyme facilitating the reaction with water, leading to the cleavage of β-lactam ring (Brahms & Peifer, 2021). The hydrolytic activity is inhibited by clavulanic acid which is a β-lactamase inhibitor than can help restore the efficacy of the antibiotics against gram-negative bacteria (Paterson & Bonomo, 2005). However, the presence of carbapenemase, an enzyme capable of hydrolyzing β-lactam antibiotics, including β-lactamase inhibitors that serve as a “last-line” defense against carbapenem antibiotics, significantly limits the treatment options for *K. pneumoniae* infections (Leavitt et al., 2009; Nordmann, Dortet & Poirel, 2012; Pitout, Nordmann & Poirel, 2015).

Of the 34 included studies, our pooled estimates indicate *armA* gene predominance, likely reflects plasmid-mediated dissemination, similar to Switzerland (Fournier et al., 2022) and UK studies (Taylor et al., 2018). Moreover, coproduction of both most predominant extended-spectrum β-lactamases (ESBL) as well as carbapenemases determinants constrains aminoglycoside and β-lactam options and reinforces MDR phenotypes. This can be explained as aminoglycoside genes were commonly plasmid-borne and often co-located with ESBL and carbapenemase determinants, enabling rapid horizontal transfer in the *K.* pneumoniae pathogen (Yang & Hu, 2022). The findings were corresponding with the other recent study where among the 96 *K. pneumoniae* isolates from Istanbul, Ankara, and Bursa carrying 16S RMTases, 34% of them coharboured an *NDM* with or without *OXA-48* or *NDM* with *KPC*, and also co-carried *CTX-M-15*, *SHV* and *TEM* (Isler et al., 2022). Similarly, a retrospective molecular epidemiology study on carbapenem-resistant *K. pneumoniae* isolates in Switzerland reported that 34 out of 72 isolates harboured several 16S RMTases such as *armA* genes (*N* = 34), *rmtF* (*N* = 25) and *rmtB* genes (*N* = 7) and among these carbapenemase-positive isolates, it co-harboured *bla*_*NDM*_ (*N* = 20) and *bla*_*KPC*_ genes (*N* = 21) (Fournier et al., 2022). Hence, the global spread of *K. pneumoniae* strains harbouring multiple resistance determinants aligns with recent WHO reports identifying carbapenem-resistant Enterobacteriaceae as critical priority pathogens (World Health Organization, 2023). The lack of harmonised surveillance systems in low- and middle-income countries may lead to underreporting of methylase-mediated resistance, masking the true burden and hampering timely response. Findings suggest a robust infection control measures and enhanced molecular AMR surveillance networks, particularly in underrepresented regions, to prevent outbreaks of multidrug-resistant organisms.

The combination of these resistance mechanisms exhibited by *K. pneumoniae* allowed it to persist in healthcare settings, complicating efforts to effectively manage infections. This can be explained where apart from highly resistant to several aminoglycosides, presence of ESBL and carbapenemase also leads to high resistance towards cephalosporin (MICs, 16–256 mg/ml) and carbapenem antibiotic groups. A review article on molecular diversity of ESBL, carbapenemases and antimicrobial resistance also revealed that they not only confer resistance towards β-lactam and carbapenem antibiotic, but also to cephems antibiotic group (Sawa, Kooguchi & Moriyama, 2020). This aligned to our findings where *K. pneumoniae* were able to degrade first, second, third and fourth generation of cephems systems possibly by the presence of β-lactamase-producing gene such as *TEM* and *SHV* and their ability to hydrolyse chemical substances containing β-lactam ring (Liakopoulos, Mevius & Ceccarelli, 2016; Bush, 2018). However, a general mechanism of ESBL and carbapenemase involves an efflux pump, reduced membraned permeability and inactivation by β-lactamases can be the contributing factors of the resistance too (Thenmozhi et al., 2014).

Nevertheless, resistance to trimethoprim-sulfamethoxazole (MICs, 4–320 mg/ml) as one of the highly reported cases is growing concern. This can be reported in this review where trimethoprim-sulfamethoxazole was classified as a folate pathway antagonist and was the second most resistant antibiotic group to *K. pneumoniae* isolates (Nafplioti et al., 2021; Spadar et al., 2021). Although trimethoprim-sulfamethoxazole antibiotic was not directly linked to the mechanism of β-lactamase enzyme, the presence of additional resistance genes such as sul1 gene (Venkatesan et al., 2023) may contribute to the synergistic effect among resistance genes in *K. pneumoniae*. The mechanism of actions when combining these two agents can create a synergistic anti-folate and helps to block bacterial biosynthesis and indirectly become bactericidal (Eyler & Shvets, 2019). This interplay between resistance determinants reveals key gaps in our understanding, as the role of these non-β-lactamase resistance mechanisms has not been explored in detail in this review.

According to World Health Organization (2023), such interrelated roles of resistance genes call for comprehensive investigations to unravel the molecular basis of additional resistance mechanisms, particularly in strains harbouring multiple resistance genes. From a clinical perspective, early detection of methylase genes could play a crucial role in guiding empiric therapy by signalling high-level aminoglycoside resistance, thereby rendering them from use before phenotypic susceptibility results were available. In such cases, alternative antimicrobial agents can be prioritized depending on the resistance profile, as continued use of ineffective agents could facilitates resistance spread and compromising future treatment options. Furthermore, molecular screening for RMTases genes can support infection control measures by enabling early identification of resistant strains, indirectly strengthening hospital surveillance systems. In the ongoing battle against antibiotic resistance, future studies should consider cooperating next-generation sequencing (NGS) technologies such as PacBio and Oxford Nanopore platforms to enhance the detection of resistance genes (Yamin et al., 2023). These long-read sequencing technologies provide a more comprehensive view of resistance by generating large, complete pathogen genome datasets, offering deeper insights into resistance mechanisms. Of 34 included studies, four researchers used WGS techniques for methylation detection. As reported by Yusof et al. (2022), most researchers have utilized sequencing and WGS methods to study the prevalence of mutations in genes associated with colistin resistance in *K. pneumoniae* strains. This trend has been fueled by the growing affordability of sequencing technology, despite the high level of expertise required for data analysis. Computational resources and bioinformatics expertise were essential for the comprehensive data analysis in this study. Therefore, it is of the utmost importance to create a bioinformatics solution that is centered on the development of straightforward analysis tools that necessitate only fundamental programming skills. Integrating artificial intelligence with bioinformatics to develop a novel pipeline for *K. pneumoniae* WGS data, specifically on resistance detection analysis, could be a valuable direction for future research. This approach would be particularly beneficial for researchers with limited programming expertise, making data analysis more accessible and efficient.

Future research should emphasize longitudinal genomic surveillance to track resistance trends over time, sequencing-based surveillance as part of routine infection control programs and detailed studies on plasmid co-carriage dynamics. Since methylase genes often coexisted with other resistance determinants on mobile genetic elements, systematic monitoring would help inform novel decolonization strategies. Experimental studies using knockout mutants could clarify the individual contribution of methylase genes to high-level aminoglycoside resistance in clinical settings. Moreover, large-scale genomic epidemiology studies integrating methylation data with plasmid tracking would help elucidate how horizontal gene transfer shapes local and global outbreaks.

## Limitation

This review article encountered several limitations. First, ethical considerations were inadequately addressed with only 23.5% of studies (eight out of 34) took ethical issues into consideration. This present a significant gap, as ethical considerations are crucial, particularly in studies involving the human subjects or clinical samples. Additionally, the absence of ethical oversight might also lead to selection bias and potential ethical violations, as samples collection might not adhered to the standardized ethical protocols. Therefore, future studies should ensure that ethical approval and informed consent are obtained when necessary to ensure the credibility of the findings. Second, methodological heterogeneity was substantial because the evidence base was dominated by PCR with limited sequencing approaches, which likely introduced ascertainment bias and affect the comparability of results across studies. Incorporating standardize workflows for detecting 16S RMTase production would mitigate this bias and better inform empirical therapy choices. Lastly, this review provides a summary of the geographical distribution of methylase genes across different countries. It shows that majority of studies were conducted in Europe and Asia, with fewer studies from North America, South America, and Australia. The overrepresentation of studies from certain regions may not accurately reflect the global distribution of these genes. This could limit the generalizability of the findings to other regions, particularly those with different healthcare systems, antibiotic usage patterns, and resistance profiles. The manuscript acknowledged the geographical bias in the included studies and discussed how this might affect the generalizability of the findings. Future reviews should aim to include studies from a wider range of regions. Although this review focuses on clinical isolates, the potential role of environmental reservoirs, livestock, and companion animals in maintaining and transmitting *K. pneumoniae* strains harboring methylase genes warrants further investigation. These genes are often plasmid-borne, facilitating horizontal gene transfer between pathogen across different hosts and ecological niches. Such mobility enables resistance determinants to disseminate not only within healthcare settings but also between animals and the environment too. A One Health approach integrating human, animal, and environmental surveillance is essential to map the full transmission dynamics and design cross-sectoral interventions. This would help identify non-clinical sources that may act as silent reservoirs, indirectly contributing to the persistence and global spread of resistance. Lastly, because this review did not include a meta-analysis, CASP appraisals were summarized descriptively and integrated narratively. This study did not conduct quality-based subgroup or sensitivity analyses, which limits the ability to quantify the influence of study quality on prevalence estimates. In particular, the potential impact of lower-quality studies on the aggregate prevalence of 16S-RMTase genes could not be quantified. Although the potential bias might distort global conclusions, this study provides an initial benchmark that highlights methodological gaps and can guide mitigation strategies such as standardized detection protocol, reporting standards and more geographically balanced surveillance particularly in under-represented regions. This highlights the need for standardized methodologies that would enable future meta-analyses.

## Conclusions

In conclusion, this review provides an initial benchmark to guide improved surveillance and standardization protocol in detecting presence of 16S rRNA methyltransferase (16S RMTase) that contributes to high resistance against aminoglycoside antibiotics. The co-existence of ESBL (extended-spectrum β-lactamase) and carbapenemases genes further escalates resistance across multiple antibiotic groups, indicating a significant challenge in treating *K. pneumoniae* infections. While computational tools for *K. pneumoniae* sequencing and application in therapeutic fields have been reviewed elsewhere, the pharmacokinetic and pharmacodynamic interactions between these agents, particularly in targeting methylase-producing bacterial strains, remain inadequately explored. Future research should focus on optimizing dosage regimens, drug synergy, and potential antagonistic effects to enhance therapeutic efficacy. Additionally, understanding the co-existence and interplay of resistance genes is crucial, as their interactions may influence drug penetration, efflux mechanisms, and enzymatic degradation, potentially leading to treatment failure. The development of novel β-lactamase inhibitors, efflux pump inhibitors, and adjuvant therapies should also be considered to enhance the effectiveness of current antimicrobial regimens. A deeper pharmacological insight into these resistance mechanisms will not only improve treatment outcomes but also aid in the surveillance and control of multidrug-resistant infections, which is essential for public health and infection management.

##  Supplemental Information

10.7717/peerj.20428/supp-1 Supplemental Information 1 The assessment quality for potential risk of bias

10.7717/peerj.20428/supp-2 Supplemental Information 2 Summary of studies on methylase genes and antibiotic resistance

10.7717/peerj.20428/supp-3 Supplemental Information 3 Classification of antibiotics groups in treating *Klebsiella pneumoniae*

10.7717/peerj.20428/supp-4 Supplemental Information 4 PRISMA Checklist

10.7717/peerj.20428/supp-5 Supplemental Information 5 Systematic Review Rationale

## Additional Information and Declarations

### Data Availability

The following information was supplied regarding data availability: This is a systematic review/meta-analysis.
