# Supplementary material for: A systematic review of the role of methylase genes in antibiotic resistance: co-existence with extended spectrum β-lactamase and carbapenemase genes in Klebsiella pneumoniae
Source: PeerJ. 2025 Dec 18;13:e20428. doi: 10.7717/peerj.20428 (PMC12718525; doi:10.7717/peerj.20428)
Supplement: Supplemental Information 1 [file peerj-13-20428-s001.docx]

**Table S1.** The Assessment Quality for Potential Risk of Bias

| Author (year) | Checklist questions | | | | | | | Score |
| --- | --- | --- | --- | --- | --- | --- | --- | --- |
|  | **1** | **2** | **3** | **4** | **5** | **6** | **7** |  |
| Yan et al (2004) | **/** | **/** | **/** | **/** |  | **/** | **/** | 6/7 |
| Lee et al (2006) | **/** | **/** | **/** | **/** |  | **/** | **/** | 6/7 |
| Bogaerts et la (2007) | **/** | **/** | **/** | **/** |  | **/** | **/** | 6/7 |
| Fritsche et al (2008) | **/** | **/** | **/** | **/** |  |  | **/** | 5/7 |
| Sabtcheva et al (2008) | **/** | **/** | **/** | **/** |  | **/** | **/** | 6/7 |
| Ma et al. (2009) | **/** | **/** | **/** | **/** |  | **/** | **/** | 6/7 |
| Wu et al. (2009) | **/** | **/** | **/** | **/** |  |  | **/** | 5/7 |
| Yu et al. (2009) | **/** | **/** | **/** | **/** |  | **/** | **/** | 6/7 |
| Tijet et al. (2010) | **/** | **/** |  | **/** |  | **/** | **/** | 5/7 |
| Zacharczuk et al. (2011) | **/** | **/** | **/** | **/** |  | **/** | **/** | 6/7 |
| Galani et al (2011) | **/** | **/** | **/** | **/** |  | **/** | **/** | 6/7 |
| Galimand et al (2012) | **/** | **/** | **/** |  |  | **/** | **/** | 5/7 |
| O'Hara et al (2013) | **/** | **/** | **/** | **/** |  | **/** | **/** | 6/7 |
| Al Sheikh et al (2014) | **/** | **/** | **/** | **/** | **/** | **/** | **/** | 7/7 |
| Belbel et al (2014) | **/** | **/** | **/** | **/** |  | **/** | **/** | 6/7 |
| Guo et al (2014) | **/** | **/** | **/** | **/** |  | **/** | **/** | 6/7 |
| Nagasawa et al (2014) | **/** | **/** | **/** | **/** | **/** | **/** | **/** | 7/7 |
| Oshiro et al (2015) | **/** | **/** | **/** | **/** | **/** | **/** | **/** | 7/7 |
| McGann et al (2016) | **/** | **/** | **/** | **/** | **/** | **/** | **/** | 7/7 |
| Piekarska et al (2016) | **/** | **/** | **/** | **/** |  | **/** | **/** | 6/7 |
| Wangkheimayum et al (2017) | **/** | **/** | **/** | **/** |  | **/** | **/** | 6/7 |
| Gopalakrishnan et al (2018) | **/** | **/** | **/** | **/** |  | **/** | **/** | 6/7 |
| Ishizaki et al (2018) | **/** | **/** | **/** | **/** |  | **/** | **/** | 6/7 |
| Taylor et al (2018) | **/** | **/** | **/** | **/** |  |  | **/** | 5/7 |
| Costello et al (2019) | **/** | **/** | **/** | **/** |  | **/** | **/** | 6/7 |
| Pakzad et al (2019) | **/** | **/** | **/** | **/** |  | **/** | **/** | 6/7 |
| Yeganeh Sefidan et al (2019) | **/** | **/** | **/** | **/** |  | **/** | **/** | 6/7 |
| Ahmadian Alashti & Ghane (2020) | **/** | **/** | **/** | **/** | **/** | **/** | **/** | 7/7 |
| Shen et al (2020) | **/** | **/** | **/** | **/** | **/** | **/** | **/** | 7/7 |
| Tipparthi et al (2020) | **/** | **/** | **/** | **/** |  |  | **/** | 5/7 |
| Nafplioti et al (2021) | **/** | **/** | **/** | **/** |  | **/** | **/** | 6/7 |
| Roch et al (2021) | **/** | **/** | **/** | **/** |  | **/** | **/** | 6/7 |
| Spadar et al (2021) | **/** | **/** | **/** | **/** | **/** | **/** | **/** | 7/7 |
| Sacco et al (2022) | **/** | **/** | **/** | **/** | **/** | **/** | **/** | 7/7 |
| Number of fulfilling each item | **34** | **34** | **33** | **33** | **8** | **30** | **34** |  |
| Percentage (%) | **100** | **100** | **97.1** | **97.1** | **23.5** | **88.2** | **100** |  |

**Quality Assessment Questions**

1. Was there a clear statement of the aims of the research?
2. Was the research design appropriate to address the aims of the research?
3. Was the execution of the index test described in sufficient detail to permit replication of the test?
4. Did the study provide a clear definition of what was considered to be a positive result?
5. If necessary, have ethical issues been taken into consideration?
6. Was the data analysis sufficiently rigorous?
7. Is there a clear statement of findings?
