# Supplementary material for: A systematic review of the role of methylase genes in antibiotic resistance: co-existence with extended spectrum β-lactamase and carbapenemase genes in Klebsiella pneumoniae
Source: PeerJ. 2025 Dec 18;13:e20428. doi: 10.7717/peerj.20428 (PMC12718525; doi:10.7717/peerj.20428)
Supplement: Supplemental Information 2 [file peerj-13-20428-s002.docx]

**Table S2***.* Summary of Studies on Methylase Genes and Antibiotic Resistance

| Author (s) | No. of *K. pneumoniae* Isolates | Country of Study | Clinical Isolates | Name of Methylase Gene | Target Site of Methylation | Antibiotics Resistance Related to Methylase Gene | Methods of Methylation Detection | Name of resistance gene (Carbapenem/ESBL) | Antibiotics Resistance Related to ESBL and Carbapenemase Genes |
| --- | --- | --- | --- | --- | --- | --- | --- | --- | --- |
| Yan et al. (2004) [35] | 21 | Taiwan | Skin and soft tissue or surgical wound, respiratory, urinary, blood | *armA (16); rmtB (5)* | 16S rRNA | Gentamicin, Kanamycin, Tobramycin, Amikacin, Trimethoprim/sulfamethoxazole | PCR & nucleotide sequencing | *blaCTX-M-14, blaCTX-M-3, blaSHV-1, blaTEM-1* | **Resistance:**  -Ciprofloxacin (MICs, >4 mg/L)  -Chloramphenicol (MICs, >256 mg/L)  -Tetracycline (MICs, >256 mg/L)  **Reduced Susceptibility:**  -Cefotaxime (MICs, 16-256 mg/L)  -Ceftazidime (MICs, 4-256 mg/L)  -Aztreonam (MICs, 4-128 mg/L)  -Cefepime (MICs, 4-64 mg/L) |
| Lee et al. (2006) [37] | 15 | Korea | Not mentioned | *armA (14) and rmtB (1)* | 16S rRNA | Arbekacin, amikacin and co-resistant with levofloxacin | PCR | *blaCTX-M-14* | **Resistance:**  -Cefoxitin  -Imipenem or Meropenem |
| Bogaerts et la. (2007) [20] | 10 | Belgium | Respiratoy, gastrointestinal, urinary, blood | *armA, rmtA, rmtB* | 16S rRNA | Gentamicin, Tonramycin, Amikacin, Netilmycin, and Kanamycin | PCR | *blaCTXM-3, blaCTXM-14, blaCTXM-15, TEM-3, SHV-like* | **Resistance:**  **-**Ceftazidime (MICs, 16 mg/ml)  -Cefotaxime (MICs, >64 mg/ml)  -Cefepime (MICs, >64 mg/ml)  -Ciprofloxacin (MICs, 1 mg/ml)  -Sulfamethoxazole/Trimethoprim (MICs, >320 mg/ml) |
| Fritsche et al. (2008) [21] | 5 | France (1); United States (1); Mexico (1); Chile (1); Argentina (1) | Not mentioned | *armA (2), rmtB (1) and rmtD (2)* | 16S rRNA | Amikacin, arbekacin, fortimicin, gentamicin, kanamycin, netilmicin and tobramycin | PCR | Not mentioned | Not mentioned |
| Sabtcheva et al. (2008) [23] | 7 | Bulgaria | Urine, blood, sputum, wound | *armA* | 16S rRNA | Gentamicin, Tobramycin, Amikacin, Kanamycin | PCR | *blaCTX-M-3, blaCTX-M-1, blaTEM-1, blaSHV-71, blaSHV-1* | **Resistance:**  -Cefotaxime (MICs, 32 mg/ml)  -Sulfamethoxazole/Trimethoprim |
| Ma et al. (2009) [36] | 92 | Taiwan | Not mentioned | *armA (44) and rmtB (37), both (1)* | 16S rRNA | Amikacin | PCR | *blaCTX-M, blaSHV* | **Resistance:**  -Ampicillin (MICs, >64 mg/ml)  -Cefazolin (MICs, 32 mg/ml)  -Cefotaxime (MICs, 64 mg/ml)  -Ceftazidime (MICs, 32 mg/ml)  -Ciprofloxacin (MICs, 4 mg/ml)  -Trimethoprim/sulfamethoxazole (MICs, 4 mg/ml) |
| Wu et al. (2009) [38] | 8 | Shanghai, China | Not mentioned | *armA (6) and rmtB (2)* | 16S rRNA | Gentamicin and amikacin | PCR and sequencing | *blaCTX-M, blaTEM-1, bla-SHV-1, blaSHV-12* | Not mentioned |
| Yu et al. (2009) [39] | 21 | Wenzhou, China | Abscess, urine, sputum, | *armA (3), rmtB (13), both armA and rmtB (5)* | 16S rRNA | Gentamicin, amikacin, tobramycin | PCR and sequencing | *blaTEM-1* and *blaCTX-M (19), blaSHV (12)* | **Highly Resistance:**  **-**Tetracycline  -Trimethoprim/sulfamethoxazole |
| Tijet et al. (2010) [49] | 4 | Argentina | Not mentioned | *rmtD1* | G1405 16S rRNA | Amikacin and gentamicin | PCR | *blaTEM, blaSHV, blaKPC, blaCTX-M* | Not mentioned |
| Zacharczuk et al. (2011) [24] | 17 | Poland | Not mentioned | *armA* | 16S rRNA | Amikacin, gentamicin and kanamycin | PCR | *blaCTX-M-3, blaTEM,* and *blaSHV* | Not mentioned |
| Galani et al. (2011) [26] | 14 | Athens, Greece | Not mentioned | *rmtB (3)* | 16S rRNA | Amikacin, gentamicin, tobramycin and netilmicin | Multiplex PCR | *blaKPC-2* & *blaOXA-10 (2)* | Not mentioned |
| Galimand et al. (2012) [22] | 218 | France | Not mentioned | *ArmA (2), RmtA (41), RmtB (42), RmtC (25), RmtD (46), RmtE (30), RmtF (1), Kmr (31)* | 16S rRNA N7 G1405 | Amikacin, gentamicin, tobramycin, rifampin, fluroquinolones, chloramphenicol | PCR | *blaOXA-1* and *blaNDM-1* | Not mentioned |
| O'Hara et al. (2013) [17] | 1 | Iraq | Intramedullary wound | *RmtH* | 16S rRNA (16S RMTase) | Gentamicin, Tobramycin, amikacin, and arbekacin | PCR and sequencing | *blaCTX-M-15, blaSHV-1, blaOXA-1* | **Resistance:**  -Ceftriaxone  -Ceftazidime  -Cefepime  -Aztreonam  -Ciprofloxacin |
| Al Sheikh et al. (2014) [34] | 92 | Saudi Arabia | Urine, blood, wounds, sputum and body fluids | *armA (34), rmtB (6), npmA (2), armA + rmtB (8), armA + rmtC (2)* | 16S rRNA | Tobramycin, gentamicin, amikacin, | PCR | *blaTEM1, blaSHV-12, blaCTX-M-14* | **Resistance:**  **-**Ciprofloxacin  -Aztreonam  -Trimethoprim/sulfamethoxazole  -Meropenem  -Imipenem  -Nitrofurantoin |
| Belbel et al. (2014) [48] | 80 | Algeria | blood, urine, pus | *armA (23)* | 16S rRNA | Amikacin, gentamicin | PCR | *blaSHV, blaTEM* and *blaCTX-M* | **Resistance:**  -Amoxicillin/clavulanic acid  -Cefoxitin  -Cefotaxime (MICs, 64-512 mg/ml)  -Ceftazidime (MICs, 4-64 mg/ml)  -Ciprofloxacin  -Trimethoprim/sulfamethoxazole |
| Guo et al. (2014) [47] | 4 | Australia | Not mentioned | *armA (3), rmtC (1)* | 16S rRNA | Amikacin, Gentamicin, Tobramycin | Real-time PCR | *NDM-1, TEM, CTX-M, OXA-30, OXA-10* | Not mentioned |
| Nagasawa et al. (2014) [41] | 15 | Japan | Not mentioned | *rmtB (5), armA (10)* | 16S rRNA | Gentamicin and amikacin | Loop-mediated isothermal amplification (LAMP) | Not mentioned | Not mentioned |
| Oshiro et al. (2015) [42] | 21 | Japan | Not mentioned | *armA (9), rmtC (2), rmtF (7), rmtC + rmtF (3)* | 16S rRNA | Arbekacin and amikacin | Immunochromatographic assay using novel monoclonal antibodies (mAbs) | Not mentioned | Not mentioned |
| McGann et al. (2016) [28] | 22 | United States (12), Germany (2), Afghanistan (4), Honduras (4) | Not mentioned | *armA (7), rmtH (6), rmtF (3), rmtC (1), rmtB (3), armA + rmtC (1)* | 16S rRNA (16S RMTase) | Arbekacin, amikacin, gentamicin and tobramycin | Real-time PCR | *blaKPC, blaNDM* | Not mentioned |
| Piekarska et al. (2016) [25] | 451 | Warszawa, Poland | Urine and wound | *armA (3)* | 16S rRNA | 4,6-disubstituted deoxystreptamines (Gentamicin, Kanamycin, Amikacin, Neomycin) | Polymerase Chain Reaction (PCR) & DNA Sequencing | Not Mentioned | Not mentioned |
| Wengkheimayum et al. (2017) [44] | 9 | India | Not mentioned | *rmtC (6), rmtB (3)* | 16S rRNA | Streptomycin, tobramycin, amikacin, kanamycin | Polymerase Chain Reaction (PCR) | *blaNDM, blaCTX-M, blaTEM, blaOXA-2* | **Resistance:**  -Cefepime  -Ceftazidime  -Ceftriaxone  -Cefixime  -Ofloxacin  -Aztreonam  -Trimethoprim/sulfamethoxazole |
| Gopalakrishnan et al. (2017) [45] | 103 | Chennai, Tamil Nadu | Urine | *armA, rmtB* | 16S rRNA | Gentamicin, Amikacin, Tobramycin | Polymerase Chain Reaction (PCR) | *blaCTXM, blaTEM, blaVIM, blaSHV, blaIMP, blaNDM-1* | Not mentioned |
| Ishizaki et al. (2018) [43] | 3 | Japan | Not mentioned | *armA, npmA* | 16S rRNA | Arbekacin, Gentamicin, Apramycin, Butirosin A | No detection test done, however the specific methylation gene were introduced onto the *Klebsiella pneumonia* strain | Not mentioned | Not mentioned |
| Taylor et al. (2018) [18] | 502 | United Kingdom and Ireland | Screening swab, urine, tissue and fluid, blood and line tip, respiratory, faecal, environmental | *armA, rmtC, rmtF* | 16S rRNA | Amikacin, Gentamicin, Tobramycin | PCR & Whole Genome Sequecing (WGS) | *blaNDM, blaOXA-48, blaKPC, blaVIM* | Not mentioned |
| Costello et al. (2019) [19] | 23 | North America, Europe, Latin America, Asia Pacific | Not mentioned | *armA, rmtB1, rmtB4, rmtF1* | 16S rRNA | Amikacin, Gentamicin, Tobramycin | PCR & Sequencing | Not mentioned | Not mentioned |
| Pakzad et al. (2019) [31] | 154 | Iran | Urine, trachea, ulcer and sputum | *armA, rmtB* | 16S rRNA | Kanamycin, Gentamicin, Tobramycin, Netilmycin and Amikacin | PCR | *blaTEM, blaSHV, blaCTX-M* | **Resistance:**  -Ceftazidime  -Cefotaxime |
| Yeganeh Sefidan et al. (2019) [32] | 57 | Azerbaijan; Northwest Iran: Tabriz & Ormiah | urine, blood, burn, wound, trachea, sputum, feces, peritoneal, and cerebrospinal fluid | *rmtC (n=8), rmtB (n=4)* | 16S rRNA | Gentamicin, Amikacin, Tobramycin, Kanamycin | Boiling method and PCR assay | Not mentioned | Not mentioned |
| Ahmadian Alashti & Ghane (2020) [33] | 100 | Tehran, Iran | urine | *rmtC (59.8%), rmtA (51.2%), rmtD (47.6%), mtF (43.9%), rmtE (41.5%), armA (41.4%), rmtB (7.3%)* | 16S rRNA | Gentamicin, Amikacin | DNA extraction using phenol-chloroform and PCR | Not mentioned | Not mentioned |
| Shen et al. (2020) [40] | 137 | China: Jiangxi (n=40), Shandong (n=19), Shanghai (n=18), Henan (n=15), Zhejiang (n=18), Hubei (n=11), Fujian (n=9), and Hunan (n=7) | bloodstreams infections (BSI) | *rmtB (89.7%), armA (6%), rmtB + armA (3%)* | 16S rRNA | Gentamicin, Amikacin | PCR and Sanger Sequencing | Carbapenem: *blaKPC, blaNDM, blaIMP* ESBL: *blaCTXM* and *blaSHV* | **Resistance:**  -Aztreonam  -Ciprofloxacin  -Imipenem  -Meropenem  -Cefoxitin  -Cefotaxime  -Cefepime  -Ceftazidime |
| Tipparthi et al. (2020) [46] | 55 | Telangana, India | Bile fluid, blood, endotracheal aspirate secretions, pus, sputum, tissue, urine and would swab | *rmtB (n=7), armA (n=4), rmtC (n=4), rmtD (n=3)* | 16S rRNA | Amikacin, Gentamicin, Netilmycin | DNA extraction and Multiplex PCR | Carbapenem: *blaKPC, blaOXA-48, blaVIM* | **Resistance:**  -Ciprofloxacin  -Ofloxacin  -Ceftazidime  -Ceftriaxone  -Cefotaxime  -Imipenem  -Meropenem  -Ceferperazone/ sulbactam  -Piperacillin/ tozabactam |
| Nafplioti et al. (2021) [27] | 42 | Athens, Greece | Blood, urine, lower respirotary tract, pus | *rmtB (n=41), armA (n=1)* | 16S rRNA | Amikacin, Gentamicin, Tobramycin, apramycin | Multiplex PCR | Carbapenem:  *blaKPC, blaVIM, blaOXA-48*; ESBL: *blaOXA-10, blaTEM, blaCTX-M* | **Resistance:**  -Ceftazidime-avibactam  -Meropenem-vaborbactam  -Trimethoprim/sulfamethoxazole  -Ciprofloxacin |
| Roch et al. (2021)  [50] | 100 | San Paulo, Brazil | Bloodstreams infection (BSI) | *rmtB* | 16S rRNA | Resistant to all 4,6-disubstitued 2-deoxystreptamine: Amikacin, Gentamicin | Whole Genome Sequencing | *blaKPC-2* | **Resistance:**  -Plazomicin |
| Spadar et al. (2021) [29] | 8 | Lisbon, Portugal | Multi-locus sequence types (MLSTs) *Kp* | *DNA adenine methylase (Dam) of the GATC motif downstreams of genes: fosA* | n6-methyladenine (m6A) | Resistant to fosfomycin | PacBio Seqeuncing | *blaCTX-M-15, blaOXA-1, blaKPC-3* | **Resistance:**  -Piperacillin/tazobactam  -Trimethoprim/sulfamethoxazole  -Imipenem |
| Sacco et al. (2022) [30] | 4 | Rome, Italy | Respiratory tract samples | *rmtc* | 16S rRNA | Aminoglycosides | PCR & Whole Genome Sequencing | *blaNDM-1* | **Resistance:**  -Amoxicillin/clavulanic acid  -Cefepime  -Cefotaxime  -Ceftaxidime  -Imipenem  -Meropenem  -Piperacillin/tazobactam  -Ceftolozone/tazobactam  -Ceftazidime/avibactam |
