# Supplementary material for: A systematic review of the role of methylase genes in antibiotic resistance: co-existence with extended spectrum β-lactamase and carbapenemase genes in Klebsiella pneumoniae
Source: PeerJ. 2025 Dec 18;13:e20428. doi: 10.7717/peerj.20428 (PMC12718525; doi:10.7717/peerj.20428)
Supplement: Supplemental Information 3 [file peerj-13-20428-s003.docx]

**Table S3.** Classification of Antibiotics Groups in Treating *Klebsiella pneumoniae*

| Antibiotics | MICs | Studies |
| --- | --- | --- |
| Cephems/Cephalosporin | | |
| *1^st^ Generation* | | |
| Cefazolin | 32 mg/ml | [36] |
| *2^nd^ Generation* | | |
| Cefoxitin | NA | [37, 40, 48] |
| *3^rd^ Generation* | | |
| Cefotaxime | 16-256 mg/ml | [20, 23, 30, 31, 35, 36, 40, 46, 48] |
| Ceftazidime | 4-256 mg/ml | [17, 20, 30, 31, 35, 36, 40, 44, 46, 48] |
| Ceftriaxone | NA | [17, 44, 46] |
| Cefixime | NA | [44] |
| *4^th^ Generation* | | |
| Cefepime | 4-64 mg/ml | [17, 20, 30, 35, 40, 44] |
| Folate Pathway Antagonist | | |
| Trimethoprim-Sulfamethoxazole/ Co-trimaxazole | 4-320 mg/ml | [20, 23, 27, 29, 34, 36, 39, 44, 48] |
| Broad Spectrum Fluoroquinolone | | |
| Ciprofloxacin | 1-4mg/L | [17, 20, 27, 34, 35, 36, 40, 46, 48] |
| Broad Spectrum Quinolone | | |
| Ofloxacin | NA | [44, 46] |
| Carbapenems | | |
| Imipenem | NA | [29, 30, 34, 37, 40, 46] |
| Meropenem | NA | [30, 34, 37, 40, 46] |
| Monobactam | | |
| Aztreonam | 4-128 mg/L | [17, 34, 35, 40, 44] |
| Phenicol | | |
| Chloramphenicol | NA | [35] |
| Tetracycline | | |
| Tetracycline | NA | [35,39] |
| Penicillin | | |
| Ampicillin | >64 mg/ml | [36] |
| Nitrofurans | | |
| Nitrofurans | NA | [34] |
| Drug Combination | | |
| *Beta-lactam and Beta-lactam Inhibitor* | | |
| Amoxicillin-clavulanate acid | NA | [30, 48] |
| Piperacillin-tazobactam | NA | [29, 30, 46] |
| Ceftazidime-avibactam | NA | [27, 30] |
| Meropenem-vaborbactam | NA | [27] |
| Ceftolozane-tazobactam | NA | [30] |
| *3^rd^ Generation Cephalosporin and Beta-lactam Inhibitor* | | |
| Cefoperazone-sulbactam | NA | [46] |
