# Supplementary material for: A systematic review of the role of methylase genes in antibiotic resistance: co-existence with extended spectrum β-lactamase and carbapenemase genes in Klebsiella pneumoniae
Source: PeerJ. 2025 Dec 18;13:e20428. doi: 10.7717/peerj.20428 (PMC12718525; doi:10.7717/peerj.20428)
Supplement: Supplemental Information 5 [file peerj-13-20428-s005.docx]

**Systematic-Review Rationale**

This systematic review entitles “**A Systematic Review of the Role of Methylase Genes in Antibiotic Resistance: Co-Existence with Extended Spectrum Beta-Lactamase and Carbapenemase Genes in Klebsiella pneumoniae**” addresses a critical and emerging area in antimicrobial resistance research by examining the role of 16S rRNA methyltransferase genes in conferring resistance in *Klebsiella pneumoniae*, and their co-existence with extended-spectrum beta-lactamase (ESBL) and carbapenemase genes. This review is intended for a multidisciplinary audience including clinical microbiologists, infectious disease researchers, molecular microbiologists, pharmacologists, healthcare policymakers, and researchers in the fields of microbial genomics, antimicrobial stewardship, and public health. By synthesizing evidence from 34 studies across various geographic regions, clinical sample types, and detection methods, this review offers a comprehensive overview of the prevalence, distribution, and resistance patterns associated with methylase genes in *K. pneumoniae*. The findings are especially relevant in guiding treatment decisions and informing infection control strategies, as the coexistence of methylase genes with ESBL and carbapenemase genes contributes to heightened resistance against several antibiotic groups such as aminoglycosides, cephalosporins, and carbapenems. Furthermore, this review also highlights the need for enhanced global surveillance and novel therapeutic approaches to address the growing threat of multidrug-resistant *K. pneumoniae*. We hope that the insights provided in this work will serve as a valuable reference for both clinical and research communities working to combat antibiotic resistance cause by *K. pneumoniae*.
